# Long Noncoding RNA Small Nucleolar Host Gene: A Potential Therapeutic Target in Urological Cancers

**DOI:** 10.3389/fonc.2021.638721

**Published:** 2021-04-22

**Authors:** Zitong Yang, Qinchen Li, Xiangyi Zheng, Liping Xie

**Affiliations:** Department of Urology, The First Affiliated Hospital, Zhejiang University School of Medicine, Hangzhou, China

**Keywords:** lncRNA, SNHG, prostate cancer, bladder cancer, kidney cancer

## Abstract

The incidence of urological cancer has been gradually increasing in the last few decades. However, current diagnostic tools and treatment strategies continue to have limitations. Substantial evidence shows that long noncoding RNAs (lncRNAs) play essential roles in carcinogenesis and the progression, treatment response and prognosis of multiple human cancers, including urological cancers, gastrointestinal tumours, reproductive cancers and respiratory neoplasms. LncRNA small nucleolar RNA host genes (SNHGs), a subgroup of lncRNAs, have been found to be dysregulated in tumour cell biology. In this review, we summarize the impacts of lncRNA SNHGs in urological malignancies and the underlying mechanisms.

## Introduction

Urological cancer is one of the most common malignancies in humans. Among the urological cancers, prostate cancer (PCa), bladder cancer (BCa) and kidney cancer are the three most common (1). Although treatment strategies have improved significantly, the recurrence rate remains high, and advanced tumour status tends to be associated with unfavourable outcomes (2–4). Therefore, much research in the last one to two decades has been dedicated to exploring potential biomarkers and identifying promising options for patients with urological malignancies.

Noncoding RNAs (ncRNAs) represent a large group of RNAs that lack the capacity to encode proteins (5). Instead, ncRNAs play important roles in epigenetic modification, transcription and translation, and the majority of ncRNAs act as regulatory factors in gene expression (6). LncRNA is a type of ncRNA longer than 200 nucleotides. lncRNAs are involved in the intracellular transportation of macromolecular substances, such as proteins, and ensure that they are delivered to appropriate functional regions and are activated or deactivated correctly (7). Recent literature rejected the former definition of lncRNA as transcription noise and revealed its multiple roles in the regulation of cell behaviour, especially during tumorigenesis and progression (8). Small nucleolar RNAs (snoRNAs) are a class of ncRNAs that predominantly exist in the nucleolus and are approximately 60-300 nucleotides in length (9). In humans, more than half of snoRNAs are transcribed from host genes, of which SNHGs account for nearly four-fifths (10). The aberrant expression of SNHGs, including lncRNA SNHGs, has been shown to be associated with the origin and development of several types of cancers (11).

Although correlations between lncRNA SNHGs and urological cancers have been reported, the roles that lncRNA SNHGs play in urological cancers are diverse and complex. We aim to comprehensively review this issue in this article.

## lncRNA SNHGs in Prostate Cancer

**Figure 1:**
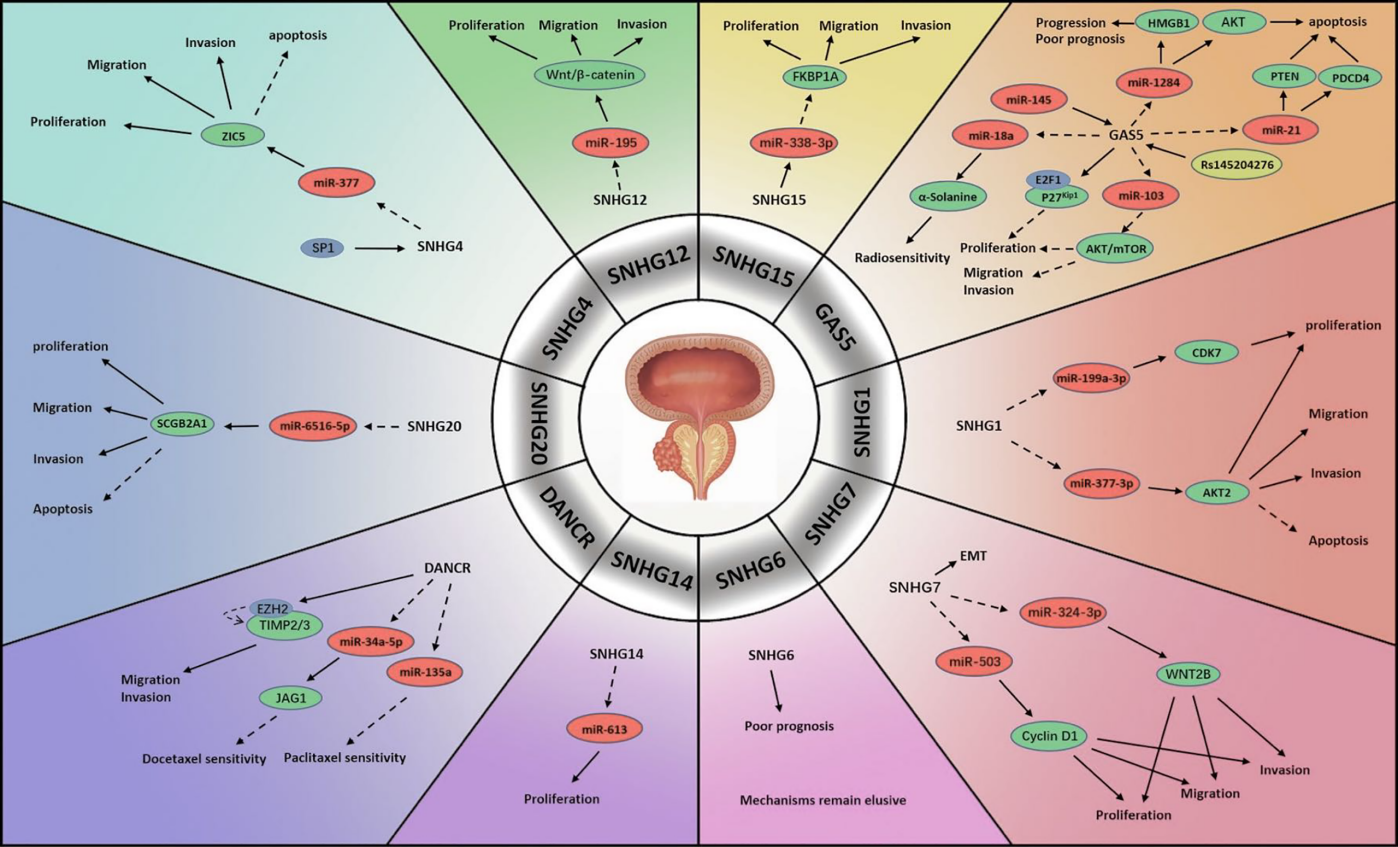
Mechanisms of lncRNA SNHGs in prostate cancer. → Stimulate, ⤏ Inhibit.

In men, PCa has long had the highest incidence of all tumours, and its incidence continues to grow globally each year (12) (**Figure 1**). Relative to the progression of other malignant tumours, the progression of PCa is slow, and many patients can achieve long-term tumour-bearing survival. However, when the disease advances to the terminal stage, i.e., castration-resistant prostate cancer (CRPC) or metastatic castration-resistant prostate cancer (mCRPC), the prognosis is unsatisfactory, and most mortality occurs in this stage (13). Thus, it is imperative to clarify the mechanism of tumorigenesis and tumour progression at the gene level and to develop novel strategies for treatment.

SNHG1 is regarded to participate significantly in the proliferation and metastasis of multiple neoplasms (14). PCa is partly attributed to the abnormal expression of SNHG1. In one study, scientists compared PCa specimens and specimens of adjacent non-tumour tissue and found that the expression level of SNHG1 was much higher in the former than in the latter. MiR-199a-3p, which was shown to be negatively associated with SNHG1 expression, was strongly repressed in PCa cells. It was suggested that SNHG1 might enhance cyclin-dependent kinase 7 (CDK7) encoding by mediating miR-199a-3p expression, which boosted cell replication (15). Another possible role of SNHG1 was also observed. MiR-377-3p, which acts as a suppressor of cell proliferation and a promoter of apoptosis, was blocked by the upregulation of SNHG1 in PCa. The downstream target was confirmed to be protein kinase B2 (AKT2), which promotes cell growth and exhibits anti-apoptosis activity. Their results suggested that the SNHG1/miR-377-3p/AKT2 pathway is linked to the progression and prognosis of PCa (16).

SNHG2, also named growth-arrest-specific-5 (GAS5), is a host gene of many snoRNAs and is widely considered a tumour suppressor in numerous human neoplasms (17). Multiple studies have reported that GAS5 is significantly correlated with the tumorigenesis, progression, clinical characteristics, treatment sensitivity and prognosis of PCa. High GAS5 expression might lead to mild clinical features of PCa. It was revealed that GAS5 was capable of repressing tumour cell viability and proliferation by blocking the protein kinase B (AKT)/mammalian target of rapamycin (mTOR) signalling pathway. In this process, miR-103 acted as the intermediate factor, which could be negatively regulated by GAS5 (18). Another pathway was believed to be associated with E2F transcriptional factor 1 (E2F1). Wherein, GAS5 attached E2F1 to the P27^Kip1^ promoter to initiate effects on the cell cycle, which decelerated PCa development (19). Pickard et al. studied the associations between GAS5 and PCa cell apoptosis and found that GAS5 served as a trigger of apoptosis in various cell lines (20). Yacqub-Usman et al. investigated the interactions between GAS5 and mTOR inhibitors in PCa and demonstrated that GAS5 reduced the tolerance of tumour cells to mTOR inhibitors (21). In a study concerning the modulation of GAS5 in PCa radiotherapy GAS5 was found able to silence miR-18a to promote α-Solanine, a novel anti-tumour molecule able to increase tumour cell sensitivity to radiation (22). A potential upstream regulator of GAS5 was elucidated to be miR-145. Researchers demonstrated that both GAS5 and miR-145 expression was greatly attenuated in PCa specimens. In contrast, enhancing miR-145 level significantly impeded tumour cell proliferation and induced apoptosis by elevating GAS5 expression. However, the intermediate regulator between miR-145 and GAS5 was not identified (23). Interestingly, some studies revealed that a genetic polymorphism in GAS5 known as rs145204276, a 5-bp indel polymorphism (-/AGGCA) in the GAS5 promoter, is involved in PCa (24–26). One demonstrated that the presence of rs145204276 markedly reduced the lymph node and seminal vesicle infiltration of PCa cells, especially among patients older than 65 years old. Furthermore, lymph vascular violation, a potential predictor of PCa biochemical recurrence, was identified to be reduced (25). The mechanism of rs145204276 activity was revealed by another, which showed that rs145204276 greatly upregulated GAS5 and stimulated its transcription. By downregulating miR-1284, activated GAS5 could inhibit high mobility group box 1 (HMGB1), which has been identified as playing a promotive role in carcinogenesis (24). Furthermore, researchers also discovered that miR-21 might be correlated with GAS5. Elevated GAS5 suppressed miR-21, targeting phosphatase and tensin homolog deleted on chromosome ten (PTEN) and programmed cell death 4 (PDCD4) and thereby inhibiting the occurrence of PCa (26). In contrast to the above observations, Zhang et al. reported GAS5 overexpression in PCa cell specimens. They demonstrated that GAS5-007, a transcript of GAS5, accelerated cell proliferation and prevented apoptosis in PCa (27).

Another focus of study is SNHG4, which has been reported to participate in several types of cell activity (28). SNHG4 has also been investigated in PCa. In a study concerning 113 PCa patients and tested their tumour tissues, a positive correlation between SNHG4 level and overall survival (OS) was observed. Further investigation showed that transcription factor SP1 activated SNHG4 upregulation, contributing to tumour cell proliferation and metastasis. Blocking SNHG4 may impair cell growth and migration and inspire apoptosis through elevating miR-377, which is regulated by SNHG1, as mentioned above. In this process, Zinc finger protein 5 (ZIC5) was identified as another downstream target of miR-377 (29).

A meta-analysis concerning SNHG6 summarized its predictive utility regarding the pathological characteristics and clinical outcomes of various neoplasms (30). In PCa, elevated expression of SNHG6 in tumour tissues was speculated to be associated with an unfavourable prognosis. The researchers constructed protein-protein interaction (PPI) networks involving SNHG6 and conducted bioinformatics analysis, which revealed the association of SNHG6 with multiple cell activities such as translation, mRNA modulation and catabolism, rRNA modification and ribosome construction. However, the signalling pathway mediating the SNHG6 functions in PCa was not clarified, a topic that warrants further investigation (31).

Accumulating studies indicate that SNHG7 might be a regulatory molecule in diverse human neoplasms, including urological tumours (32). Researchers have revealed various mechanisms by which SNHG7 regulates tumour cell activities in PCa. Augmented levels of SNHG7 suppressed miR-324-3p and then activated the Wingless-type MMTV integration site family member 2b (WNT2B) in PCa. The epithelial-mesenchymal transition (EMT) was also found to be involved in this pathway, accelerating metastatic process (33). Another approach by which SNHG7 regulates PCa involves the SNHG7/miR-503/Cyclin D1 pathway. SNHG7 depletion inhibited PCa cell replication and growth and froze the cell cycle at the G0/G1 phase. In particular, SNHG7 reduced the miR-324-3p level, which negatively regulates the Cyclin D1 protein (34). Furthermore, SNHG7 might be an independent risk factor that patients with higher SNHG7 levels were more likely to have an unfavourable outcome than those with lower SNHG7 levels (35).

SNHG12 is a dysregulated molecule in the pathogenesis, progression and prognosis of diverse neoplasms (36). In a study, SNHG12 was markedly increased in PCa specimens, energizing tumour cells. SNHG12 activated the miR-195/Wnt/β-catenin pathway, which promoted the pathogenesis of PCa (37).

SNHG13, a lncRNA also known as differentiation antagonizing non-protein coding RNA (DANCR), plays a regulatory role as an oncogene in multiple human cancers. DANCR expression is correlated with pathogenesis and progression and is predictive of the outcome of several neoplasms (38, 39). PCa studies have indicated that DANCR may affect the treatment response to chemotherapy. One literature reported an advanced DANCR level in docetaxel (DTX) nonresponsive cell lines and that DANCR depletion improved the effects of DTX. MiR-34a-5p, extensively sponged by DANCR, was capable of impairing Jagged 1 (JAG1) encoding, which is associated with chemotherapy tolerance (40). The role of DANCR in paclitaxel treatment was also revealed that the upregulation of DANCR inhibited the expression of miR-135a, which could sensitize tumour cells in paclitaxel chemotherapy (41). In another study, the impairment of DANCR in PCa cell lines abrogated cell viability and mobility. In that work, DANCR functioned similarly to SNHG2, downregulating TIMP metallopepitidase inhibitor 2/3 (TIMP2/3) by activating the binding of enhancer of zeste homolog 2 (EZH2) to the promoter of TIMP2/3, which accelerated PCa metastasis. Furthermore, activation of the androgen-androgen receptor (AR) pathway attenuated the DANCR level, decelerating PCa progression (42).

A novel oncogene, SNHG14 exerts regulatory effects in lung cancer, digestive cancer, reproductive cancer, haematological cancer and osteosarcoma (43). SNHG14 is markedly overexpressed in PCa tumour cells. Impeding SNHG14 could greatly suppress cell replication capacity. MiR-613 was found to be blocked by SNHG14 in PCa. Furthermore, silencing SNHG14 led to a reduced PCa tissue volume compared with that in a control group. This research established the mechanism by which SNHG14 promotes PCa through miR-613 (44).

Studies have revealed SNHG15 as a regulator in the progression of several human malignancies (45). Scientists examined its status and modes of function in PCa, finding an aberrant increase in SNHG15 in tumour cells relative to normal tissues. SNHG15 impairment was found to compromise tumour cell activity and reduce tumour aggressiveness. They further revealed miR-338-3p as a target of SNHG15, the downstream molecule of which was found to be FKBP prolyl isomerase 1A (FKBP1A). They revealed the mechanism involving the SNHG15/miR-338-3p/FKBP1A pathway and showed that SNHG15 promoted FKBP1A by negatively regulating miR-338-3p in PCa cells (46).

SNHG20 plays crucial roles in tumorigenesis and progression in several human cancers, including gastrointestinal cancer, liver cancer, breast cancer, cervical cancer, oral cancer and urological cancer (47). Both PCa tissues and cell lines demonstrated greatly elevated SNHG20 levels in one study. Enhancing SNHG20 level activated anti-apoptosis function and accelerated cell propagation. The mechanism was reported to involve the SNHG20/miR-6516-5p/secretoglobin family 2A member 1 (SCGB2A1) signalling pathway: SNHG20 suppressed miR-6516-5p level, which negatively modulated SCGB2A1, a positive regulator of tumour cell activity that is elevated in PCa (48).

## lncRNA SNHGs in Bladder Cancer

**Figure 2:**
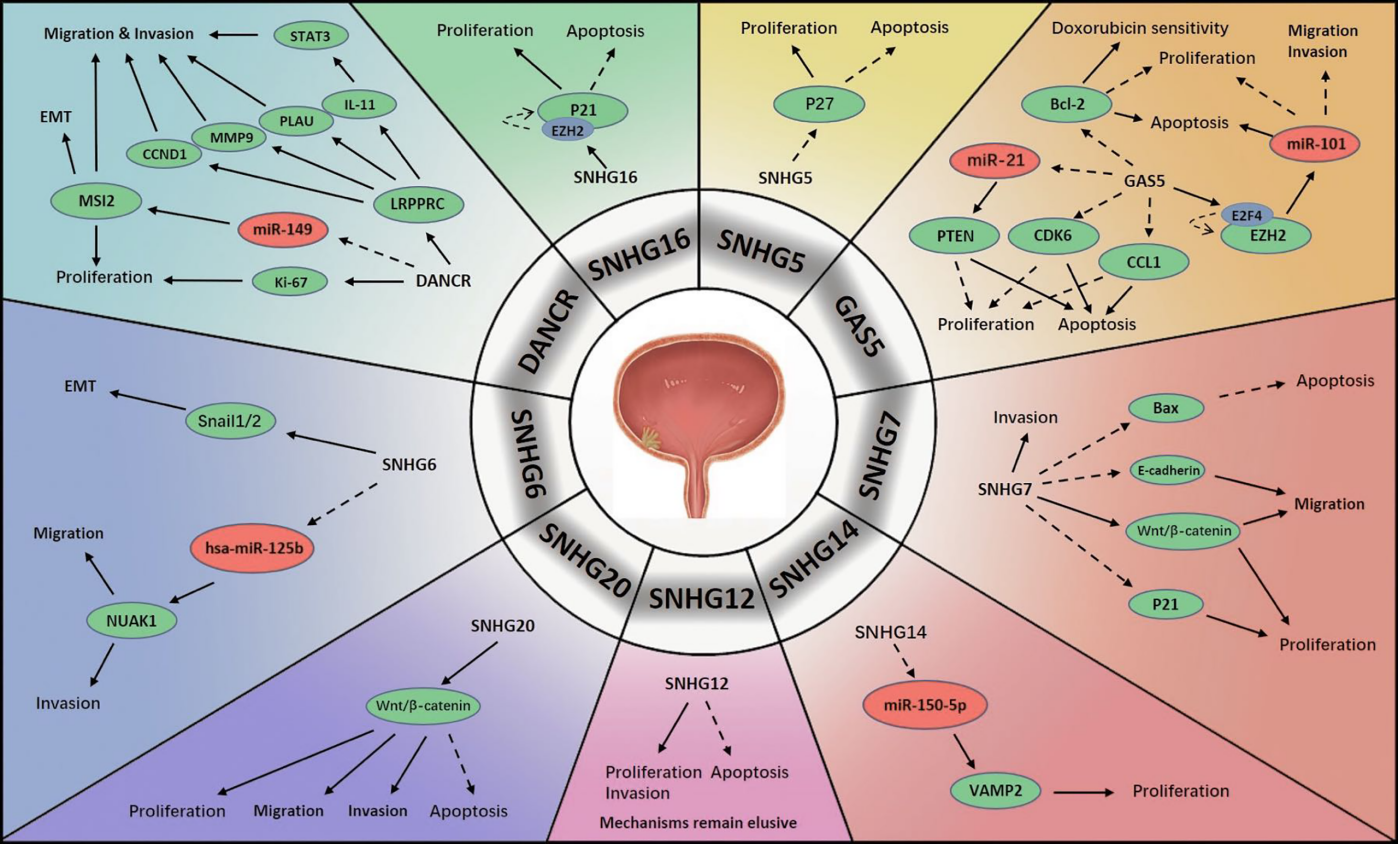
Mechanisms of lncRNA SNHGs in bladder cancer →. Stimulate ⤏, Inhibit.

BCa is another common concern around the globe; although it does not rank as one of the top cancers in incidence or mortality rate, over 400 000 patients are diagnosed every year (49) (**Figure 2**). Urinary ultrasound combined with cystoscopy is very effective for detection. Transurethral resection and intravesical chemotherapy are the most recommended treatments for non-muscle invasive bladder cancer (NMIBC). However, a considerable number of patients face high risks of recurrence and progression, especially when routine surveillance is absent (50). Once NMIBC advances to muscle invasive bladder cancer (MIBC), the treatments become limited to partial or radical cystectomy and systemic chemotherapy (51). Generally, to prevent recurrence, patients diagnosed with BCa are likely to receive a long period of medical follow up involving procedures such as cystoscopy, which is mentally and physically unpleasant for patients. Therefore, it is crucial to clarify the genetic mechanisms of BCa carcinogenesis, progression and recurrence to identify biomarkers for disease management. The discovery of lncRNA SNHGs may provide clinical options.

An extensive decrease in GAS5 level in BCa cells has been found in multiple studies. Similar to PCa, BCa has been shown to be associated with activation of the GAS5/miR-21/PTEN signalling pathway (52). GAS5 was confirmed to inhibit tumour cell proliferation and promote cell apoptosis, and these processes were modified by cyclin-dependent kinase 6 (CDK6), which had a negative correlation with GAS5 in BCa progression (53). Similar correlations between GAS5 and BCa were also discovered that GAS5 impaired the encoding of B-cell lymphoma-2 (Bcl-2), which is an anti-apoptosis factor in carcinogenesis. Apart from this, GAS5 enhanced the response of BCa cell lines to doxorubicin chemotherapy (54). Another literature demonstrated that GAS5 initiated the binding of E2F transcriptional factor 4 (E2F4) with the EZH2 promoter, thereby exerting its proapoptotic function. The prognostic value of GAS5 was also evaluated, and BCa with elevated GAS5 expression tended to display lower malignancy than that with lower GAS5 expression (55). Another possible pathway was explored that GAS5 repressed BCa cell proliferation and activated apoptosis by sponging chemokine ligand 1 (CCL1), a regulatory factor in BCa, which might increase cell viability (56). Additionally, GAS5 was found to be of great value in the diagnosis and prognosis of BCa (57). Avgeris et al. identified GAS5 as an independent predictor of BCa prognosis, especially for NMIBC. In BCa, the absence of GAS5 was associated with more rapid recurrence. Furthermore, GAS5 was suggested to potentially be predictive of disease aggressiveness, and lower levels were found to be associated with higher malignancy (58). Intriguingly, scientists measured lncRNA levels in urine samples and constructed a lncRNA panel consisting of two lncRNAs: GAS5 and uc004cox.4. Analysis revealed that GAS5 had superior diagnostic performance to conventional urine cytology (57).

SNHG5 plays a pivotal role in diverse human neoplasms (59). Unsurprisingly, it also plays an important role in urological malignancies. SNHG5 has been found to be greatly overexpressed in BCa specimens relative to normal urothelial specimens. In addition, increased SNHG5 has been shown to be associated with a more malignant tendency in several aspects, such as tumour burden, aggressiveness and pathological features. Experiments have shown that knocking down SNHG5 has antiproliferative and proapoptotic effects. Additionally, SNHG5 has been shown to exert its tumour-promoting function through the sponging mRNA p27, which is reduced in BCa cells (60).

The regulatory mechanisms of SNHG6 in BCa were revealed. Scientists found that SNHG6 was aberrantly upregulated in BCa tissues, which stimulated EMT by negatively regulating E-cadherin encoding and positively regulating vimentin encoding. Snail1/2 was suggested to modulate this activity. As expected, SNHG6 greatly enhanced the aggressiveness of BCa cells, and silencing SNHG6 reduced BCa cell viability. MiR-125b was subsequently identified as an intermediate molecule of SNHG6, and a negative feedback circle between SNHG6 and hsa-miR-125b in BCa was revealed as SNHG6/hsa-miR-125b/NUAK Family Kinase 1(NUAK1) signalling in BCa promotion (61).

The elevated expression of SNHG7 in BCa was also observed, but the mechanisms varied. Chen et al. repressed SNHG7, which resulted in reduced survival, growth and migration of tumour cells and cell cycle arrest; these findings confirmed that SNHG7 accelerates BCa progression. Moreover, it was verified that Wnt/β-catenin pathway was stimulated by SNHG7 to activate carcinogenesis (62). Others found that SNHG7 depletion greatly reduced cell viability, migration and invasion and triggered cell apoptosis. Possible targets of SNHG7 were revealed to be Bax, p21 and E-cadherin, which were previously demonstrated to initiate apoptosis, modulate the cell cycle and impede cell proliferation, and block cell migration, respectively (63).

One study demonstrated that SNHG12 was abnormally regulated in BCa. Although the mechanism was not identified, they showed that silencing SNHG12 may hamper cell proliferation and invasion and exert proapoptotic effects in BCa. Additionally, they reported that the overexpression of SNHG10 and SNHG12 might lead to worse relapse-free survival (RFS) in BCa (64).

DANCR expression is aberrantly upregulated in BCa pathogenesis and progression. DANCR was considered capable of stimulating lymph node infiltration and metastasis by elevating the levels of interleukin 11 (IL-11), plasminogen activator urokinase (PLAU), matrix metalloproteinase 9 (MMP9), and CCND1(or cyclin D1). In addition, leucine-rich pentatricopeptide repeat containing (LRPPRC), which is differentially expressed between BCa cells and normal cells, was shown to maintain the mRNA structures of these four molecules (65). When DANCR was silenced in BCa cells, cell viability and mobility were markedly compromised. The mechanism involved the upregulation of musashi RNA binding protein 2 (MSI2) by DANCR *via* the repression of miR-149. High levels of MSI2 were found to be positively associated with the malignant degree of BCa (66).

Researchers discovered that SNHG14 expression was elevated in BCa cells compared with non-tumour cells, which suggested a poorer prognosis. The signalling pathway involved in BCa was the SNHG14/miR-150-5p/vesicle-associated membrane protein 2 (VAMP2) axis. MiR-150-5p acted as a downstream target of SNHG14, and its upregulation antagonized the promoting effects of SNHG14. Moreover, miR-150-5p was found to reduce VAMP2 level, thereby enhancing cell activity and facilitated BCa progression (67).

SNHG16 is a novel factor active in various human cancers and predictive of poor prognosis (68). Scientists detected an abnormally overexpressed SNHG16 level in serum exosomes of BCa patients. SNHG16 was expressed to a greater extent in MIBC than in NMIBC, suggesting that it stimulates tumour progression (69). Similarly, others found that SNHG16 level was greatly elevated in the nuclei of BCa cells. The upregulation of SNHG16 was confirmed to be associated with tumour severity and detrimental to clinical outcomes. When silencing SNHG16, cell growth was delayed and apoptosis was accelerated. The possible target of SNHG16 was p21, which was previously demonstrated to interfere with the cell cycle, maintaining it at G1 stage (70).

SNHG20 is another important oncogene in BCa. Researchers observed a pronounced increase of SNHG20 in BCa specimens and that patients with higher SNHG20 levels tend to have less favourable outcomes. They found that SNHG20 deficiency reduced the viability and aggressiveness of tumour cells and stimulated apoptosis. Furthermore, the impairment of SNHG20 impeded activation of the Wnt/β-catenin pathway and thereby inhibited tumour progression (71).

## lncRNA SNHGs in Kidney Cancer

**Figure 3:**
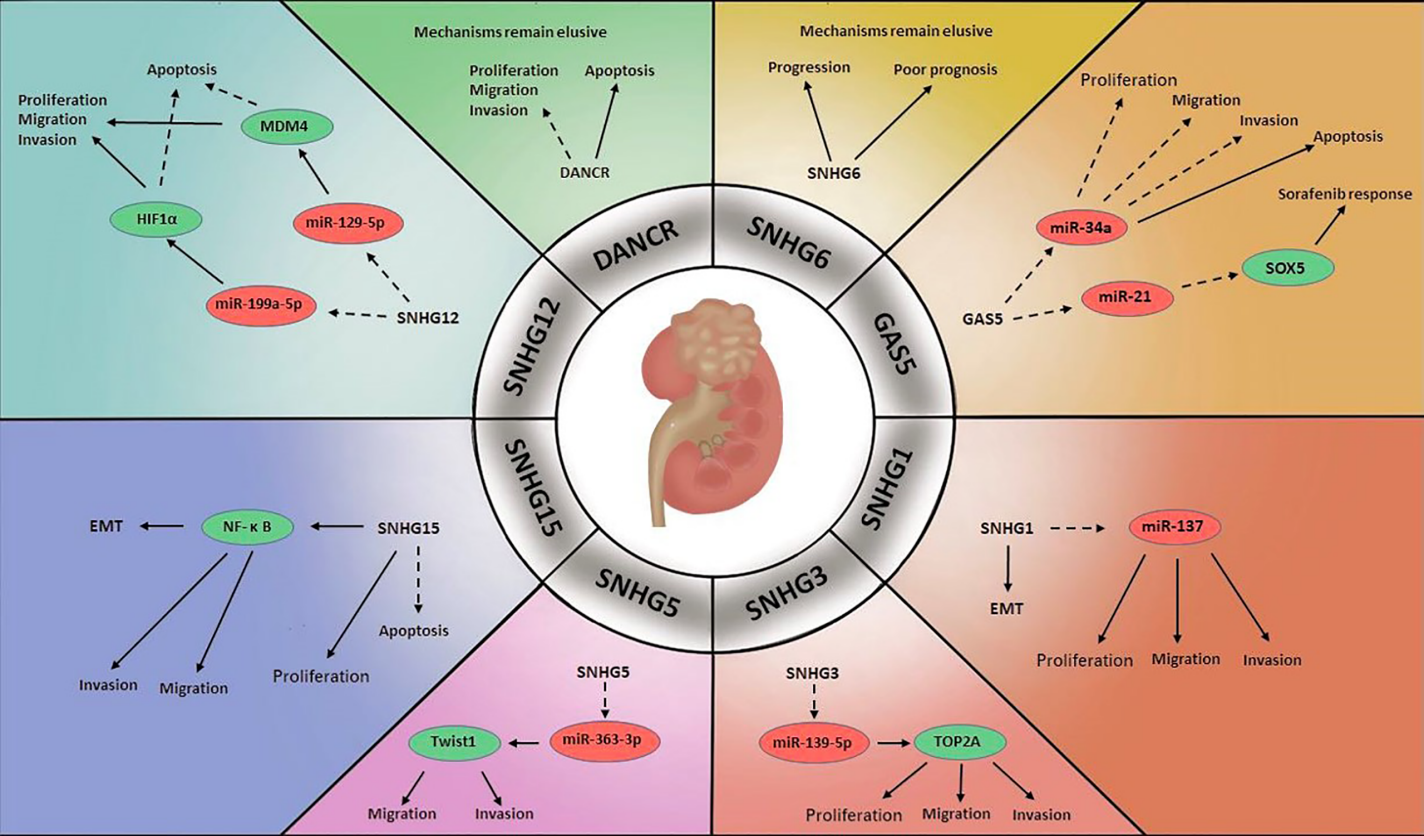
Mechanisms of lncRNA SNHGs in renal cell carcinoma →. Stimulate ⤏, Inhibit.

The incidence of kidney cancer is slightly lower than that of bladder cancer, accounting for approximately 2% of human cancers (72) (**Figure 3**). Renal cell carcinoma (RCC) accounts for most kidney cancers, the predominant and most malignant subtype of which is clear cell renal cell carcinoma (ccRCC) (73). As RCC does not respond well to chemotherapy and radiotherapy, surgical resection has been the most common treatment strategy (74). However, the prognosis remains unsatisfactory and lower than that of BCa and PCa. Thus, research to identify promising biomarkers to improve the diagnosis and treatment of RCC is urgently needed. Several lncRNA SNHGs have been shown to be associated with RCC.

In one study, SNHG1 was found to be greatly upregulated in RCC cells relative to surrounding non-tumour cells, and SNHG1 inhibition was shown to block the EMT, thereby reducing tumour cell growth and invasion. It was speculated that miR-137 was a target of SNHG1, as SNHG1 knockdown promoted miR-137 expression in RCC cells (75).

Research on RCC has identified GAS5 as a tumour suppressor. Toraih et al. measured GAS5 and miR-34a levels in RCC specimens, and the results suggested that miR-34a was upregulated in the former and downregulated in the latter. Although the mechanism was not clarified, the authors speculated that there might be reciprocal regulation between GAS5 and miR-34a in oncogenesis (76). Others observed a large decrease in GAS5 level in RCC cell lines relative to normal cell lines. They found that enhanced GAS5 expression inhibited cell proliferation, migration and invasive ability and triggered cell apoptosis (77). Liu et al. demonstrated that GAS5 could improve the treatment response to sorafenib in RCC. GAS5 level was markedly increased in sorafenib-sensitive patients compared with sorafenib-resistant patients. They reported that GAS5 and miR-21 interacted with each other and that the target of miR-21 was sex determining region Y-box protein 5 (SOX5), which was negatively modulated by miR-21 (78). GAS5 may be of outstanding prognostic value for RCC clinical features, especially in the prediction of advanced stage and capsular invasion (79).

The dysregulation of SNHG3 has been another focus (80). SNHG3 is overexpressed in tumour specimens of ccRCC, which was significantly correlated with unfavourable clinical and pathological features. SNHG3 depletion has been shown to inhibit cell viability, proliferation and aggressiveness by releasing the negative regulatory effects of miR-139-5p on Topoisomerase II Alpha (TOP2A), identified as the downstream factor of miR-139-5p, and stimulating tumour progression (81).

The role that SNHG5 plays in ccRCC was considered as an oncogene. Scientists revealed that SNHG5 was greatly overexpressed in ccRCC specimens compared with adjacent specimens. Silencing SNHG5 expression activated apoptosis and compromised the invasive capability of ccRCC cells by upregulating miR-363-3p. Further study revealed that the target molecule of miR-363-3p was Twist1, which greatly enhanced cell aggressiveness (82).

SNHG6 has been confirmed to be associated with the clinical features and prognosis of RCC. Elevated SNHG6 expression in RCC specimens were markedly associated with RCC tumour burden and lymph node infiltration. Moreover, SNHG6 was identified as a risk factor in RCC prognosis that patients with higher SNHG6 levels were more likely to have lower OS (83).

An abnormally elevated SNHG12 level in ccRCC was observed. Upregulation of SNHG12 predicted an advanced stage of tumour while knockdown of SNHG12 hampered tumour progression and stimulated apoptosis. SNHG12 was found to exert its regulatory effects through downregulating miR-129-5p, the target of which is murine double minute 4 (MDM4), which has been reported to be overexpressed in several cancers, including ccRCC (84). Another signalling pathway involved this molecular was that *via* sponging miR-199a-5p, SNHG12 promoted transcription factor HIF1α to accelerate RCC carcinogenesis (85).

In contrast to observations in PCa and BCa, DANCR level was found to be reduced in RCC specimens. Enhanced DANCR expression sharply impeded cell growth and aggressiveness and promoted apoptosis in RCC cells. Although the mechanism was not revealed, these findings suggested that DANCR might act as a tumour inhibitor in RCC (86).

In RCC, increased SNHG15 expression was found to facilitate RCC progression, while SNHG15 depletion resulted in proapoptotic cells and cell cycle arrest. The detailed mechanism was that SNHG15 stimulated the EMT through regulating NF-κB, ultimately enhancing RCC proliferation, migration and invasion (87).

Yang et al. measured lncRNA SNHGs levels in ccRCC and found that SNHG1, GAS5, SNHG3-8, SNHG11, SNHG12, SNHG15-17, SNHG20, SNHG22 and SNHG25 were overexpressed in RCC cells compared with healthy renal cells. In contrast, SNHG9, SNHG10, DANCR and SNHG14 levels were decreased in RCC cells. Among these SNHGs, SNHG3 and SNHG15 exhibited strong prognostic performance. Both were negatively correlated with OS in RCC, and SNHG15 alone was associated with RFS. Additionally, compared with localized RCC, advanced RCC tended to be associated with higher levels SNHG3 and SNHG15 (88).

Wilms’ tumour (WT), or nephroblastoma, is the most common renal malignancy in children, accounting for nearly 7% of all cancers (89, 90). A literature reported the discovery of abnormally increased SNHG6 levels in WT tissues. Impairment of SNHG6 reduced tumour cell viability and mobility and stimulated apoptosis. MiR-15a was confirmed to negatively interact with SNHG6, with the downregulation of SNHG6 leading to enhanced miR-15a expression, which repressed the TGF-β-activated kinase 1 (TAK1)/c-Jun-N-terminal kinase (JNK) and Wnt/β-catenin signalling pathways. Both of these pathways were considered to stimulate tumour progression (91).

## Conclusion

Generally, lncRNA SNHGs engage in the modulation of cancer biology in two ways. In one way, lncRNA SNHGs act as competing endogenous RNAs (ceRNAs) that occupy the binding sites between microRNA (miRNA) and its target genes, preventing the inhibitive effects of miRNA on transcription level (92, 93). In the other way, lncRNA SNHGs regulate certain signalling pathways involved in protein encoding and influence tumour pathogenesis and progression on protein expression level. LncRNA SNHGs that significantly involved in urological cancers include SNHG1, GAS5, SNHG3-7, SNHG12, DANCR, SNHG14-16 and SNHG20 (**Table 1**).

**Table 1:** LncRNA SNHGs in urological cancers.

SNHG	Chromosomal location	Main subcellular location		Related urological cancer types	Related regulatory molecules and(or) pathways	Related tumor biologies	Role	References
SNHG1	11p12.3	Cytoplasm		PCa, RCC	MiR-199a-3p/CDK7, miR-377-3p/AKT2, miR-137	Proliferation, migration, invasion, apoptosis, EMT	Oncogene	(15, 16, 75)
GAS5 (SNHG2)	1q25.1	Cytoplasm		PCa, BCa, RCC	MiR-103/AKT/mTOR, miR-18a/α-Solanine, miR-1284/HMGB1, miR-21/PTEN, miR-21/PDCD4, miR-21/SOX5, E2F4/EZH2, E2F1/P27, miR-145, miR-34a, CDK6, CCL1	Proliferation, migration, invasion, apoptosis	Oncogene/antioncogene (GAS5-007)	(18, 20–24, 26, 27, 52–56, 58, 77, 78)
SNHG3	1p35.3	Cytoplasm		RCC	MiR-139-5p/TOP2A	Proliferation, migration, invasion	Oncogene	(81)
SNHG4	5q31.2	Cytoplasm		PCa	MiR‐377/ZIC5	Proliferation, migration, invasion	Oncogene	(29)
SNHG5	6q14.3	Cytoplasm		BCa, RCC	P27, miR-363-3p/Twist1	Proliferation, cell cycle, migration, invasion, apoptosis	Oncogene	(60, 82)
SNHG6	8q13.1	Cytoplasm		PCa, BCa, RCC, WT	Hsa‐miR‐125b/NUAK1, miR-15a/TAK1/JNK, miR-15a/Wnt/β-catenin signalling	Proliferation, migration, invasion, EMT	Oncogene	(31, 61, 83, 91)
SNHG7	9q34.3	Cytoplasm	PCa, BCa		MiR-503/cyclin D1, miR-324-3p/WNT2B, Wnt/β-catenin signaling	Proliferation, cell cycle, migration, invasion, EMT	Oncogene	(33–35, 62)
SNHG12	1p35.3	Cytoplasm	PCa, BCa, RCC		MiR195/Wnt/β‐catenin signaling, miR-199a-5p/HIF1α, miR-129-5p/MDM4	Proliferation, migration, invasion, apoptosis	Oncogene	(37, 64, 84, 85)
DANCR (SNHG13)	4q12	-	PCa, BCa, RCC		MiR-34a-5p/JAG1, miR-149/MSI2, EZH2/TIMP2/3, miR-135a, IL-11, PLAU, MMP9, CCND1	Proliferation, migration, invasion, apoptosis	Oncogene (PCa, BCa)/antioncogene (RCC)	(40–42, 65, 66)
SNHG14	15q11.2	Cytoplasm	PCa, BCa		MiRNA-150-5p/VAMP2, miR-613	Proliferation, cell cycle	Oncogene	(44, 67)
SNHG15	7p13	Cytoplasm	PCa, RCC		MiR-338-3p/FKBP1A, NF-κB signaling	Proliferation, cell cycle, migration, invasion, EMT	Oncogene	(46, 87)
SNHG16	17q25.1	Nucleus	BCa		P21	Proliferation, cell cycle, migration, invasion, apoptosis	Oncogene	(70)
SNHG20	17q25.2	Cytoplasm	PCa, BCa		MiR-6516-5p/SCGB2A1, Wnt/β-catenin signaling	Proliferation, migration, invasion, apoptosis	Oncogene	(48, 71)

The differential expression of lncRNA SNHGs in tumour and non-tumour cells, as well as the discovery of underlying mechanisms by which they function have proved its non-negligible role in tumorigenesis, progression and prognosis of multiple human cancers. In urological cancers, most of lncRNA SNHGs act as an oncogene. While interestingly, DANCER (SNHG13) serves as an oncogene in PCa and BCa but an antioncogene in RCC. Similar phenomenon also exists in GAS5 (SNHG2). The reason why these two molecules play opposite roles in different urological cancers needs further clarification. For sure, the comprehensive and in-depth understanding of the biological features of lncRNA SNHGs may provide us more clues in the exploration of novel biomarkers and potential therapeutic targets in the managements of urological cancers.

## Author Contributions

ZY and XZ conceived the structure of manuscript. XZ, LX, ZY and QL critically revised the manuscript. ZY and QL drafted initial manuscript and made the figures and table. All authors contributed to the article and approved the submitted version.

## Funding

This study was supported by grants from the National Natural Science Foundation of China (81972374 and 81772744).

## Conflict of Interest

The authors declare that the research was conducted in the absence of any commercial or financial relationships that could be construed as a potential conflict of interest.
